# Addressing Parameter Variability in Corneal Biomechanical Models: A Stepwise Approach for Parameters’ Optimization

**DOI:** 10.3390/biomimetics10100683

**Published:** 2025-10-10

**Authors:** José González-Cabrero, Carmelo Gómez, Manuel Paredes, Francisco Cavas

**Affiliations:** 1 Department of Structures, Construction and Graphical Expression, Technical University of Cartagena, 30202 Cartagena, Spain; jose.gonzalezc@edu.upct.es (J.G.-C.); carmelo.gomez@upct.es (C.G.); 2 Bioengineering and Applied Computational Simulation Research Group, Technical University of Cartagena, 30202 Cartagena, Spain; 3 ICA, Université de Toulouse, UPS, INSA, ISAE–SUPAERO, MINES–ALBI, CNRS, 3 rue Caroline Aigle, 31400 Toulouse, France; paredes@insa-toulouse.fr

**Keywords:** corneal biomechanics, Holzapfel model, inflation test, hyperelastic material, parameters estimation

## Abstract

Biomechanical modeling of the cornea is crucial for understanding the progression of some ocular diseases and optimizing surgical treatments. However, hyperelastic non-linear material models, such as those used for corneal tissue, often yield highly variable parameter sets in the scientific literature, influenced by factors like the chosen optimization intervals and differences between tensile and inflation test curve optimization, both of which are addressed in this study. This variability complicates the understanding of corneal mechanical properties. In this research, the aim is to optimize and calibrate the key parameters of the corneal material model, particularly focusing on $c_{1}$, $c_{2}$, $k_{1}$ and $k_{2}$, using the Holzapfel–Gasser–Ogden (HGO) hyperelastic model, and a novel methodology is proposed that separately estimates the isotropic and anisotropic components in a stepwise manner, addressing the issue of multiple parameter sets fitting experimental curves similarly. This approach helps to standardize corneal material models and improve the reliability of parameter estimations. Moreover, accurate biomechanical characterization within this framework contributes not only to clinical applications but also to biomimetics, inspiring the design of artificial corneal substitutes and bioengineered materials.

## 1. Introduction

The human eye receives over 90% of external sensory information, highlighting the critical importance of ocular health for quality of life [1]. The cornea, contributing approximately two-thirds of the eye’s refractive power [2], plays a vital role in this process. This avascular structure, with an average thickness of 550 μm centrally and 750 μm peripherally, is primarily composed of stroma, which accounts for 90% of its thickness and consists of collagen fibers embedded in a proteoglycan matrix and arranged in a region-specific orientation [3].

The cornea’s mechanical behavior is commonly modeled using anisotropic hyperelastic models [4,5], essential to optimize surgical outcomes and understanding diseases like ectasia, characterized by structural weakening of collagen fibers [6]. Hyperelastic models, such as the Gasser–Holzapfel–Ogden (GHO) and Holzapfel–Gasser–Ogden (HGO) models, have become standard in corneal studies due to their capacity to describe anisotropic properties and fiber dispersion [7,8,9,10]. These models assume near-incompressibility and provide parameters representing isotropic stiffness, fiber stiffness, and non-linear deformation. However, parameter variability across studies, influenced by differences in experimental techniques, tissue properties, and mathematical simplifications, complicates direct comparisons and the reliability of conclusions between studies [11,12,13,14].

Tensile and inflation tests are the primary experimental methods to estimate corneal parameters. However, the two approaches yield inconsistent results due to differences in testing conditions and the mathematical models applied. For example, studies using tensile tests, such as those by [11,12,13], have reported wide variability in parameter values, while inflation tests, as demonstrated by [14,15], highlight the sensitivity of material parameters to experimental conditions.

Experimental comparisons have shown that strip extensometry and inflation produce markedly different responses [16] and that multiple loading scenarios are critical for accurate parameter identification [17]. Moreover, recent advances such as Brillouin microscopy have enabled non-invasive biomechanical characterization [18], while age- and IOP-related collagen recruitment studies further emphasize the variability in corneal mechanical response [19]. Recent ex vivo and animal studies also demonstrate discrepancies between uniaxial and biaxial testing outcomes [20], highlighting the difficulty of establishing universally valid parameter sets.

Variations in geometric measurements, non-uniformity of corneal properties, and model-specific assumptions contribute to these discrepancies. Furthermore, high correlations between experimental data and model predictions (high correlation coefficients $R^{2}$) do not always ensure unique or physically valid parameter sets. This issue arises when parameter compensations occur, leading to multiple sets that fit experimental data similarly. Consequently, it becomes necessary to establish robust methods for parameter estimation that minimize variability and improve reliability.

This study aims to address these challenges by (1) developing a method to characterize corneal material parameters of the HGO model through a novel stepwise optimization approach that separates the estimation of the isotropic ground-matrix components from the anisotropic fiber parameters to reduce variability and ensure physical consistency in the estimated parameters; (2) proposing parameter bounds and optimization algorithms to demonstrate the high variability inherent in the characterization problem, driven by the large number of parameter combinations that yield similarly high correlation with the experimental curve.

Beyond its clinical relevance, this research also fits within a biomimetic perspective. The cornea, combining transparency, anisotropy, and mechanical resilience, serves as an inspiring model for bioengineered materials. Advances in contact lenses, tissue substitutes, and artificial corneal constructs seek to replicate these properties, highlighting how biomechanical modeling can guide the design of biomimetic solutions that emulate their structural and functional efficiency. In this context, an accurate characterization of the cornea’s hyperelastic properties is essential to ensure that such designs achieve physiological-like behavior.

## 2. Materials and Methodology

In this research, a detailed methodology is implemented to optimize the material parameters of corneal tissue ($c_{1}$, $c_{2}$, $k_{1}$ and $k_{2}$) using tensile and inflation tests within the Holzapfel–Gasser–Ogden (HGO) model framework [7]. This model is adopted because it combines physiological realism with simplicity: it separates the isotropic ground matrix ($c_{1}$, $c_{2}$) from collagen–fiber reinforcement and reproduces the cornea’s characteristic exponential stiffening ($k_{1}$, $k_{2}$). Other models, such as the five-parameter Mooney–Rivlin formulation [21], can also capture pronounced nonlinearity; however, they make it more difficult to distinguish which parameters govern the initial, matrix-dominated segment of the curve and which govern the later, fiber-driven non-linear response.

**Figure 1:**
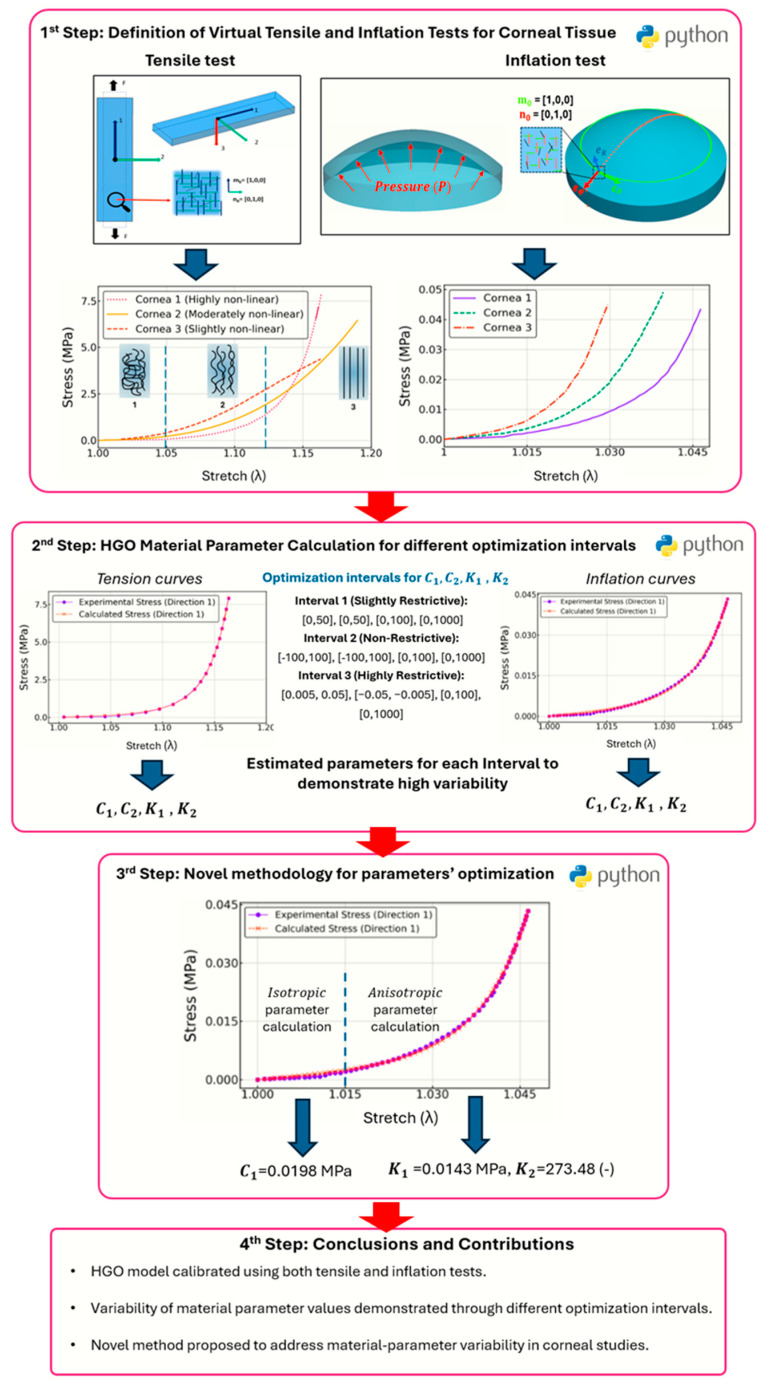
The four steps that comprise the material parameter estimation analysis and the contributions of the study.

Both tensile and inflation tests are mathematically defined to simulate the cornea’s mechanical behavior under physiological conditions. The methodology is summarized in the scheme shown in Figure 1.

Python version 3.11.9 (Python Software Foundation) is used for data analysis, parameter optimization, and curve fitting. Core libraries were NumPy for array algebra, SciPy (differential_evolution, best/1/bin) for parameter optimization, and Matplotlib for plotting.

In the optimization process, a differential evolution algorithm is employed to iteratively adjust the material parameters ($c_{1}$, $c_{2}$, $k_{1}$ and $k_{2}$) to minimize the difference between the predicted stress values ($\sigma_{1}$, $\sigma_{2}$, $\sigma_{3}$) and the experimental data.

This approach, known as least-squares minimization, aims to reduce the total error in the predicted stress values. Penalties are also introduced to avoid physically meaningless solutions, such as negative stress values ($\sigma_{1}\leq 0$), ensuring that the parameters remain physically valid throughout the optimization process. The coefficient of determination, $R^{2}$ is used to evaluate the quality of the fit between the model’s predictions and the experimental data; it measures how much of the variance in the experimental data is captured by the model, with a value close to 1 indicating a strong fit.

The optimization analysis is conducted by implementing three different parameter bounds to demonstrate that various parameter sets can achieve high experimental fits with $R^{2}$ values close to 1, while still maintaining physical validity in order to explain the variability of parameter sets commonly reported in the literature. The goal is to analyze which parameter interval is preferable, ranging from highly restrictive, moderately restrictive, to non-restrictive bounds. Only different intervals are set for $c_{1}$ and $c_{2}$, while maintaining for $k_{1}$, and $k_{2}$. The optimization process is iterated within these intervals until the set that minimizes the error is found.

Experimental data for the tensile and inflation tests’ curves are obtained from published investigations. To mitigate inconsistencies and enhance parameter stability, a stepwise optimization approach is proposed in Section 3.3. First, $c_{1}$ is optimized for small deformations dominated by isotropic behavior. Once $c_{1}$ is established, $k_{1}$ and $k_{2}$ are optimized to capture the anisotropic non-linear behavior at larger deformations, while $c_{2}$ is neglected. This sequential approach simplifies the optimization process and reduces parameter variability, addressing a major limitation in prior studies. By first fitting $c_{1}$ on the small-strain, matrix-dominated segment and then fitting $k_{1}$ and $k_{2}$ on the non-linear, fiber-dominated segment, the estimation is split into lower-dimensional subproblems, which removes many compensating parameter combinations and thereby reduces parameter variability even when $R^{2}$ remains high.

### 2.1. Biomechanical Modeling of Corneal Material

In hyperelastic material modeling, the local deformation is described by the deformation gradient $F\;=\;\frac{\partial x}{\partial X}$, which maps a material point from its reference position X to its current deformed position x. Its determinant J = det(F) is the local volume ratio; for incompressible response, *J* = 1 (volume preservation). The Cauchy stress tensor is written as

(1) $$\sigma =-pI+\frac{2}{J}F\frac{\partial \psi }{\partial C}F^{T},$$

where σ is the Cauchy stress tensor, p is a Lagrange multiplier that enforces incompressibility and represents the hydrostatic/volumetric part of the stress, and I is the identity tensor (unit tensor) that produces an equal normal contribution in every direction. The symbol ψ denotes the strain–energy density function. The tensor ***C*** = $F^{T}F$ is the right Cauchy–Green deformation tensor, which measures strain in the reference configuration. The derivative $\frac{\partial \psi }{\partial C}$ is the tensor of partial derivatives of the energy with respect to ***C***. In practice, ***ψ*** is often split into a volumetric term and a deviatoric (shape-changing) term. In this work, incompressibility is enforced by the Lagrange multiplier p, so the volumetric contribution is represented by the hydrostatic term −pI in Equation (1); therefore, ***ψ*** in Equation (1) denotes only the deviatoric strain-energy density. The deviatoric component of ψ in the HGO model is as follows:

(2) $$\psi_{deviatoric}=c_{1}\;\left({\bar{I}}_{1}-3\right)+c_{2}\;\left({\bar{I}}_{2}-3\right)+\frac{k_{1}}{2k_{2}}\sum_{i=4,6}(exp[{k_{2}({\bar{I}}_{i}-3)}^{2}]-1)\;,$$

where $c_{1}$+ $c_{2}$ defines shear modulus, $k_{1}$ describes fiber stiffness, and $k_{2}$ accounts for nonlinearity in large strains. The invariants are $I_{1}\;=\;\mathrm{tr}\;(C)$, $I_{2}\;=\;\frac{1}{2}\;\left({\left(\mathrm{tr}\;(C)\right)}^{2}-\mathrm{tr}(C^{2})\right)$, $I_{4\;}={\;m}_{0}Cm_{0}$ and $I_{6}\;=\;n_{0}Cn_{0}$, where $m_{0}=\left[1,0,0\right]$ and $n_{0}=\left[0,1,0\right]$ are initial fiber directions.

Assuming incompressibility and following the theory of continuum mechanics, the Cauchy stress tensor can be expressed in terms of the invariants and the left Cauchy–Green deformation tensor $b=FF^{T}$ through the following expression:

(3) $$\sigma =\;-pI+\frac{2}{J}\left[\left(\frac{\partial \psi }{\delta I_{1}}+I_{1}\frac{\partial \psi }{\delta I_{2}}\right)b-\frac{\partial \psi }{\delta I_{2}}b^{2}\;+\frac{\partial \psi }{\;\delta I_{4}}m⨂m\;+\;\frac{\partial \psi }{\delta I_{6}}n⨂n\right],$$

where $m=Fm_{0}$ and $n=Fn_{0}$ are the orientation vectors in the preferred directions in the deformed configuration, and I is the identity matrix.

#### Tensile Test

During the tensile test, the tissue is clamped at both ends, and a tensile force is applied along the length in the 1-direction, resulting in elongation characterized by the stretch ratio $\lambda_{1}$ > 1. To maintain volume due to incompressibility, the tissue contracts in directions 2 and 3 perpendicularly. The deformation gradient tensor is given by

(4) $$F=\left[\begin{array}{ccc} \lambda_{1} & 0 & 0\\ 0 & \lambda_{2} & 0\\ 0 & 0 & \lambda_{3} \end{array}\right],$$

where $J\;=\;1\;=\;\lambda_{1}\lambda_{2}\lambda_{3}$ ensures incompressibility. For a tensile load in the 1-direction, with the tissue confined to the 1–2 plane, ${\lambda_{3}\;{=\;(\lambda }_{1}\lambda_{2})}^{-1}$ and $\lambda_{2}=\frac{1}{\sqrt{\lambda_{1}}}$. The Cauchy stress tensor is expressed as follows:

(5) $$\sigma \;=\left[\begin{array}{ccc} \sigma_{1} & 0 & 0\\ 0 & \sigma_{2} & 0\\ 0 & 0 & \sigma_{3} \end{array}\right]$$

**Figure 2:**
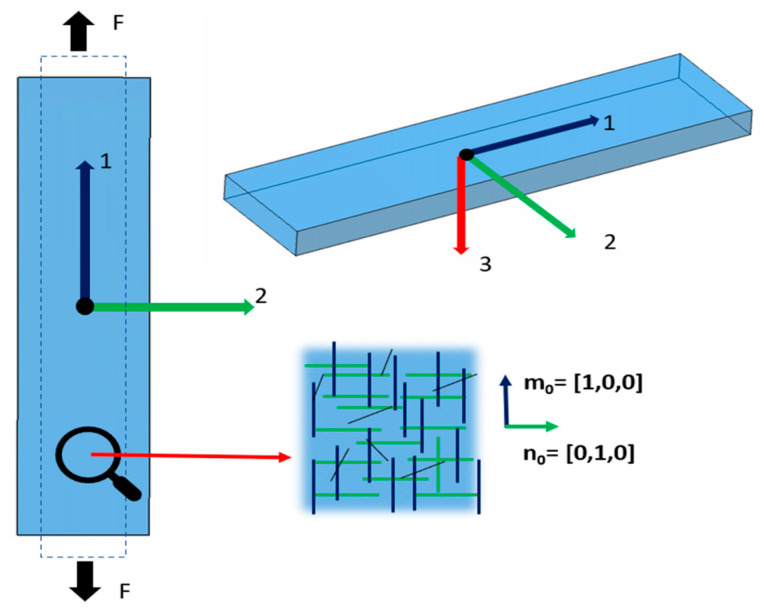
Schematic image of a tensile test.

For uniaxial tensile tests, stress is applied only in the 1-direction, so $\sigma_{1}=$ σ; $\sigma_{2}=\sigma_{3}=0$. Figure 2 illustrates the virtual tensile setup where the specimen is clamped at both ends and loaded along direction 1.

Tensile strips are typically cut along the inferior–superior or nasal–temporal anatomical axes; these are widely considered the principal corneal fiber directions in the central stroma of the cornea; to generalize across published datasets, this orientation is assumed for all cases. Accordingly, two preferred in-plane fiber families are defined and taken orthogonal in the reference configuration: $m_{0}=\left[1,0,0\right]$ aligned with the loading direction and $n_{0}=\left[0,1,0\right]$ aligned transversely. The local axis {1, 2, 3} is also indicated in the figure.

**Figure 3:**
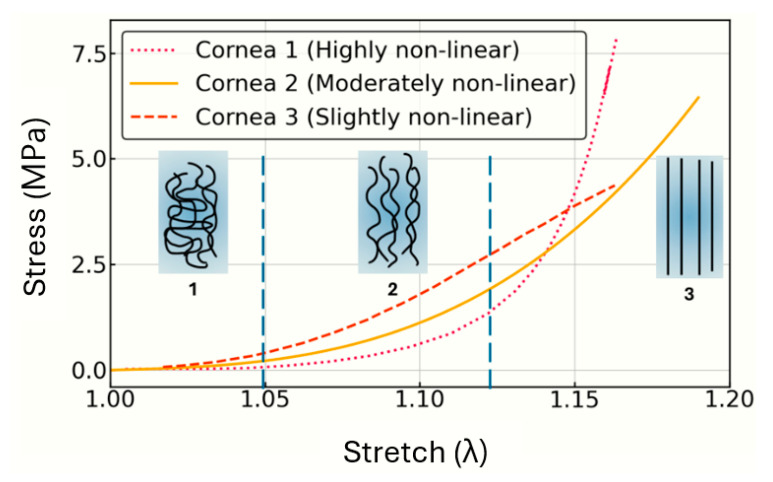
Schematic image of three tensile test curves.

The schematic Figure 3 represents the stress–stretch curves used in this research for three different corneal specimens under tensile testing obtained from [13,14], highlighting varying degrees of non-linearity in their mechanical responses. Additionally, it illustrates the collagen fiber alignment during the stretching process in three stages.

### 2.2. Inflation Test

The inflation test is critical to understand the cornea’s biomechanics, as it closely replicates in vivo conditions by applying pressure similar to intraocular pressure (IOP). Unlike uniaxial tensile tests, it provides insights into the realistic mechanical response of corneal tissue under physiological conditions. Note that an axisymmetric thin-membrane assumption is adopted to match the experimental inflation setup and as a test simplification, implying equibiaxial loading ($\sigma_{1}=\sigma_{2}=\sigma ;\;\sigma_{3}=0$). The cornea, however, is not a perfect spherical cap and exhibits regional variation and fiber dispersion; these aspects are acknowledged in Section Limitations of the Study. The deformation gradient tensor for the inflation test is

(6) $$F=\left[\begin{array}{ccc} \lambda_{1} & 0 & 0\\ 0 & \lambda_{2} & 0\\ 0 & 0 & \lambda_{3} \end{array}\right],$$

where λ_1_, λ_2_, and λ_3_ are the stretches in the meridional $e_{\varphi }$, circumferential $e_{\theta },$ and radial $e_{R}$ directions, respectively. Due to incompressibility, $\lambda_{1}$ = $\lambda_{2}$ and $\lambda_{3}$ = $1/\lambda_{1}^{2}$, with $J=\lambda_{1}^{2}\lambda_{3}=1$.

**Figure 4:**
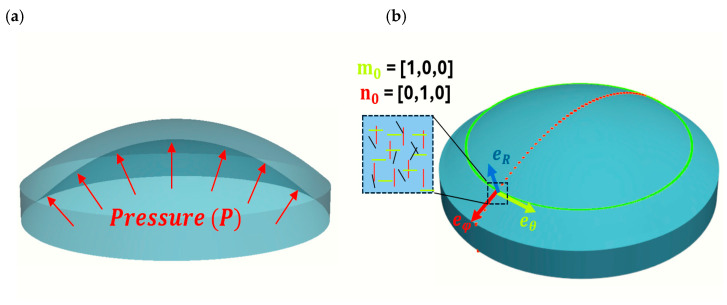
Schematic figure of an inflation test. (**a**) Applied pressure. (**b**) Meridional $e_{\varphi }$, circumferential $e_{\theta }$ and radial directions $e_{R}$. Fiber directions towards meridional $n_{0}$ and circumferential $m_{0}$.

Assuming a thin-membrane model, the cornea is treated as sufficiently thin to neglect through-thickness stresses and bending effects in the radial direction $\sigma_{R}$, resulting in a biaxial stress state in the meridional $\sigma_{\varphi }$ and circumferential $\sigma_{\theta }$ directions. The stress tensor is as follows:

(7) $$\sigma =\left[\begin{array}{ccc} \sigma & 0 & 0\\ 0 & \sigma & 0\\ 0 & 0 & 0 \end{array}\right],$$

where σ represents the stress in both directions. This setup allows studying corneal behavior under applied pressure, as shown in Figure 4.

**Figure 5:**
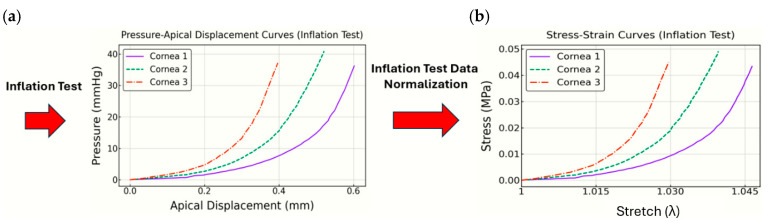
Three corneal curves of the inflation test. Conversion from pressure–apical displacement (**a**) into stress–stretch (**b**).

The equations to convert pressure–apical displacement data into stress–stretch relationships are detailed in Appendix A according to [22]. These include the conversion of pressure units, calculation of the radius of curvature and stretch, and the application of Laplace’s law to determine corneal stress. The equations account for geometric changes in the cornea during deformation, assuming it behaves as a spherical shell. Figure 5 shows the transformation of the curves [10] before and after applying the normalization process.

### 2.3. Parameter Optimization for Tensile and Inflation Tests

The four parameters ($c_{1}$, $c_{2}$, $k_{1}$ and $k_{2}$) are fitted simultaneously using the differential evolution algorithm (strategy best/1/bin), implemented in Python via SciPy (from scipy.optimize import differential_evolution). This algorithm efficiently handles complex, non-linear problems typical of hyperelastic material modeling. The optimizer minimizes a least-squares error between model predicted and experimental stresses, augmented with large penalties to enforce test physics: penalties avoid negative stress, and non-monotonic axial stress as stretch increases.

The algorithm runs up to 10,000 iterations with a tolerance of 1 × 10^−6^ on the objective. Parameter bounds are implemented as box constraints; their restrictiveness is varied to demonstrate that different intervals can yield different parameter sets, with further discussion provided in Section 3. Goodness of fit is reported with the coefficient of determination $R^{2}=1-\frac{{SS}_{Res}}{{SS}_{Tot}}$ (where ${SS}_{Res}$ is the residual sum of squares (predicted vs. experimental), and ${SS}_{Tot}$ is the total variance in experimental data).

Section 3.3 introduces a novel stepwise approach seeking to stabilize parameter identification by separating the estimation of the isotropic part ($c_{1}$) from the anisotropic part ($k_{1}$ and $k_{2}$).

## 3. Results

### 3.1. Parameter Optimization: Tensile Test

In the optimization process, three bound intervals are tested (Interval 1 (slightly restrictive), Interval 2 (non-restrictive), and Interval 3 (highly restrictive)) to demonstrate how bound choices affect the resulting parameter sets.

The rationale for the bounds selection is explanatory (to show how bounds affect identifiability), keeping the fiber terms effectively unrestrictive ($k_{1}$ in [0, 100] MPa and $k_{2}$ in [0, 1000]) while varying only the isotropic bounds. Interval 1 (“slightly restrictive”) uses positive-only ranges for $c_{1}$ and $c_{2}$ (e.g., [0, 50] MPa); whether the upper limit is 50 or 100 MPa is immaterial, provided the range is sufficiently large to calculate without restriction. Interval 2 (“non-restrictive”) allows $c_{1}$ and $c_{2}$ in [−100, 100] MPa, admitting negative $c_{2}$ values, sometimes reported to compensate $c_{1}$; any solutions with $c_{1}$ + $c_{2}$ ≤ 0 are deemed physically inadmissible even if the statistical fit is high. Interval 3 (“highly restrictive”) adopts narrow literature-based ranges around typical $c_{1}$ and $c_{2}$ values to illustrate how tight priors can over-constrain the fit and promote parameter compensation in $k_{1}$ and $k_{2}$.

Interval 1 (Slightly Restrictive) provides the most reliable results, with parameter bounds of [0, 50] for $c_{1}$ and $c_{2}$, and [0, 100] and [0, 1000] for $k_{1}$ and $k_{2}$, respectively. This interval ensures physical validity, avoiding negative shear stress values ($c_{1}$ + $c_{2}$). The algorithm consistently assigns $c_{2}$ near zero, simplifying the isotropic component to a single parameter ($c_{1}$), which effectively captures the material’s initial stiffness. This interval results in high $R^{2}$ values while maintaining interpretability, with parameter values reflecting the stiffness and fiber behavior across different non-linearities of the corneal tissue.

Interval 2 (Non-Restrictive), with bounds of [−100, 100] for $c_{1}$ and $c_{2}$, leads to unrealistic results, such as negative shear stress, rendering the parameters physically invalid despite high $R^{2}$ values. This demonstrates that overly broad bounds can cause overfitting, producing results that are statistically accurate but lack meaningful representation of material properties.

Interval 3 (Highly Restrictive) tightly constrains $c_{1}$ and $c_{2}$ to [0.005, 0.05] and [−0.05, −0.005], respectively, while maintaining the same bounds for $k_{1}$ and $k_{2}$ as in Interval 1. Although this interval produces physically valid results, it forces some parameters to their boundary values, leading to compensatory adjustments in $k_{1}$ and $k_{2}$. While useful in specific scenarios where parameter ranges are well-defined, such tight constraints may limit the optimization process and risk over-constraining the system.

Results of the estimated HGO material parameters for the optimization intervals are shown in Table 1.

**Figure 6:**
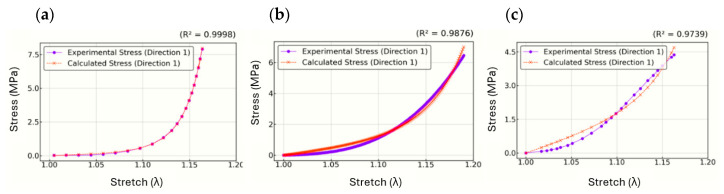
Stress–stretch tensile curves (highly non-linear (**a**), moderately non-linear (**b**), and slightly non-linear (**c**)).

Figure 6 presents the stress–stretch curves for Interval 1 (Slightly Restrictive). Plot (a) corresponds to the highly non-linear curve, (b) represents the moderately non-linear curve, and (c) shows the slightly non-linear curve. It illustrates the correlation between the experimental stress and the predicted stress. The high $R^{2}$ values (ranging from 0.97 to 0.99) demonstrate an excellent fit between the experimental and calculated curve, confirming the accuracy of the optimization process.

The three datasets fitted in this section correspond to the literature tensile curves shown previously in Figure 3 obtained from [13,14]. Stresses in directions 2 ($\sigma_{2}$) and 3 ($\sigma_{3}$) are expected to remain zero, as required by the boundary conditions of a uniaxial tensile test, confirming that the parameter set obtained from Interval 1 produces physically meaningful and reliable results across all directions.

### 3.2. Parameter Optimization: Inflation Test

Results from the inflation test are visually represented in Figure 5b, where three corneas (Cornea 1, Cornea 2, and Cornea 3) are displayed. These curves reflect a typical stress–stretch behavior, showing increasing stiffness as stretch progresses. It can be observed that the sudden non-linear behavior, or “knee,” occurs at different points along the curves, with some corneas showing an earlier rapid increase in stiffness, while others present a more gradual transition.

Cornea 1 exhibits a gradual increase in stress at lower strains, followed by a sharper rise later on. This indicates that Cornea 1 shows delayed fiber stiffening, as it is dominated by a lower initial stiffness, influenced mainly by a lower $c_{1}$. Cornea 2, on the other hand, displays a more intermediate response, with a smoother balance between early-stage stiffness and later non-linear behavior. Cornea 3 presents the sharpest increase in stress almost from the beginning of the test, which suggests that Cornea 3 has a higher initial stiffness (likely due to a higher $c_{1}$) and reaches the non-linear fiber-dominated region earlier than the others.

As in Section 3.1, the parameter optimization for the inflation test is carried out for three different intervals, from which the material parameters are optimized using the differential evolution algorithm. A detailed analysis of the set of parameters obtained for each interval and the strong correlation with the experiments are shown in Table 2, with $R^{2}$ values close to 1.

Interval 1 (Slightly Restrictive) emerges as the most suitable for parameter estimation due to its ability to provide physically meaningful values without encountering negative or unrealistic results. However, as shown in Table 2, all curves in this interval exhibit zero values for both $c_{1}$ and $c_{2}$, effectively neglecting the isotropic contribution to the cornea’s material behavior. This simplification suggests that the model primarily captures anisotropic stiffening due to fiber alignment, which may not fully represent the cornea’s initial stiffness, as the isotropic matrix plays a critical role in early deformation responses. This limitation highlights the need for further refinement in optimizing $c_{1}$, as addressed in Section 3.3.

In contrast, Interval 2 (Non-Restrictive) allows for a broader exploration of parameter values but yields physically invalid results. For example, Cornea 2 shows $c_{1}$ = 2.905 MPa and $c_{2}$ = −3.792 MPa, resulting in a negative shear modulus ($c_{1}$ + $c_{2}$), which is inconsistent with the physical interpretation of the corneal material. Despite achieving high $R^{2}$ values, these results lack physical coherence and cannot reliably describe the cornea’s mechanical properties.

Interval 3 (Highly Restrictive), while yielding physically valid results, imposes excessively tight constraints that limit the optimization process. Parameters like $c_{1}$ are consistently forced to boundary values, such as $c_{1}$ = 0.005 MPa, restricting the model’s ability to explore the cornea’s true mechanical behavior. Although this interval may be useful in specific scenarios, its restrictive nature makes it less suitable for general applications.

**Figure 7:**
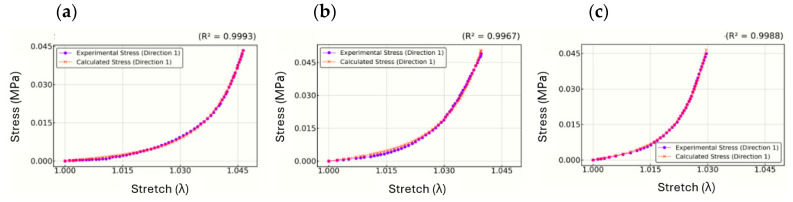
Stress–stretch inflation curves (highly non-linear (**a**), moderately non-linear (**b**), and slightly non-linear (**c**)).

Figure 7 illustrates the stress–stretch curves for the inflation test using parameters from Interval 1, with results presented for highly non-linear (a), moderately non-linear (b), and slightly non-linear (c) cases. The model accurately captures the anisotropic mechanical behavior of the cornea under inflation conditions. In plots (a, b, c), the predicted stress aligns closely with the experimental data, achieving high $R^{2}$ values ranging from 0.9967 to 0.9993. The circumferential and meridional stresses are equivalent, consistent with the equibiaxial tension assumption, while the radial stress remains near zero, as expected under thin membrane conditions. These findings confirm that the parameter set derived from Interval 1 provides a reliable and consistent representation of the cornea’s mechanical response.

### 3.3. Novel Methodology for Parameter Optimization in Corneal Inflation Tests

During the optimization process for the inflation test, it became clear that the values of $c_{1}$ were unrealistically low, being calculated as zero in some configurations. This is physically incorrect because $c_{1}$ is responsible for the isotropic stiffness of the corneal matrix, which plays a crucial role in the initial response to deformation. To address this issue, a strategy is implemented to refine the model and ensure more accurate values for $c_{1}$.

The initial portion of the stress–stretch curves, where the response is approximately linear and dominated by the isotropic matrix, is isolated and optimized separately. This step allowed the model to focus on adjusting $c_{1}$, ensuring that the early stiffness of the cornea is accurately captured. The exact cutoff for this linear segment can vary between curves, but a practical guideline is that collagen fibers begin to bear a significant share of the load at roughly 2% strain [20]. Therefore, the 0–2% strain range can be treated as approximately linear and dominated by the isotropic matrix.

After isolating the initial phase of the curves, strict limits close to zero are imposed on $k_{1}$, and $k_{2}$ to refine the optimization of $c_{1}$. It is important to remember that $c_{2}$ can be neglected to simplify the material model, reducing it to a system with only three parameters to optimize. The final values for $c_{1}$ are in Table 3, demonstrating a clear increasing trend from Cornea 1 to Cornea 3. This validates the method, as the graphs show a corresponding increase in initial stiffness, aligning with the expected mechanical behavior.

Once $c_{1}$ is fixed, the anisotropic parameters $k_{1}$ and $k_{2}$ are optimized to capture the non-linear behavior of the cornea. The resulting values reflecting the influence of fiber alignment and stiffening in the later stages of the stress–strain response are shown in Table 4:

Upon analyzing $k_{1}$, there appears to be no clear trend across the curves (it increases from Curve 1 to Curve 2 but then decreases in Curve 3). This pattern suggests that $k_{1}$ does not solely govern the non-linear behavior of the cornea and likely interacts with both $c_{1}$ and $k_{2}$, complicating its interpretation. In contrast, $k_{2}$ shows a clear increasing trend from Curve 1 to Curve 3, playing a more dominant role in the non-linear stiffening behavior observed at higher strains. This makes sense, as $k_{2}$ is closely linked to fiber stiffening. The overlap of $k_{1}$’s effects with both $c_{1}$ and $k_{2}$ complicates the optimization, reinforcing the importance of first optimizing $c_{1}$ to separate isotropic behavior from fiber-driven non-linearity. Once $c_{1}$ is fixed, $k_{1}$ and $k_{2}$ can be optimized more effectively.

In summary, $c_{1}$ increases steadily from Curve 1 to Curve 3, reflecting greater initial stiffness. $k_{1}$ follows a non-monotonic pattern, with no clear trend, while $k_{2}$ consistently rises, capturing the sharp increase in stiffness due to fiber alignment at larger strains, aligning with typical non-linear stiffening seen in the cornea during inflation tests.

## 4. Discussion

The significant variability observed in corneal biomechanical studies, particularly in tensile and inflation tests, arises from various causes. In this research, the authors highlight that these discrepancies mainly occur because different parameter sets can still make physical sense and achieve strong correlations with experimental data. This introduces challenges in defining a universally accepted set of material parameters for corneal tissues.

In tensile tests, a wide range of parameter values is reported across different studies (see Table 5). The parameters $c_{1}$, $c_{2}$, $k_{1}$, and $k_{2}$ show considerable variability, reflecting the anisotropic stiffening behavior of the cornea under uniaxial loading conditions. For example, $c_{1}$ values range from 0.0025 MPa [13] to 15.11 MPa [10] (Hoeltzel et al., 1992), illustrating a great difference in the isotropic stiffness parameter. Similarly, $k_{1}$ spans from 0.0338 MPa [15] to 103.5 MPa [12] (M. Nambiar et al., 2022), showing a wide range in fiber alignment’s contribution to corneal stiffness. $k_{2}$ values vary from 5.74966 [13] to 1280.07 [15], highlighting a massive disparity in scale in the non-linear stiffening behavior of collagen fibers across the studies. These variabilities may also contribute to geometrical differences in the corneal specimens, testing different corneal regions (central vs. peripheral), variations in experimental conditions, and the methods used to model the anisotropic behavior of the tissue.

To aid comparison across sources, Table 5 (tensile) and Table 6 (inflation) use (−) to indicate values not reported. The differences across studies reflect model choices. Some studies use an HGO formulation with a two-parameter Mooney–Rivlin isotropic base ($c_{1}$ and $c_{2}$), others report only a single isotropic parameter ($c_{1}$), and some fit their curves with a pure two-parameter Mooney–Rivlin model without fiber terms $k_{1}$ and $k_{2}$. For Table 6, note that [15] present a re-fit in which $k_{1}$ and $k_{2}$ are recalculated after fixing $c_{1}$ to the mean across specimens (0.004 MPa).

Inflation tests present distinct challenges. Variability in inflation studies is also very high (see Table 6). Apart from the previously mentioned possible sources of variability, an additional source arises from converting pressure–apical displacement curves into stress–stretch curves, which relies heavily on geometrical measurements such as the corneal base radius, thickness, and apex height. If these are measured inaccurately, they can drastically alter the resulting curves. Consequently, variation in material parameters in inflation tests stems from differences in these geometric inputs, in addition to model assumptions and experimental conditions. For instance, $c_{1}$ values in inflation tests range from 0.00291 MPa [15] to 0.32 MPa [10]. Similarly, $k_{1}$ values vary between 0.0217 MPa [15] and 0.055 MPa [10], and $k_{2}$ ranges from 291.28 [15] to 750 [10].

Tensile and inflation test parameters are not directly comparable. The mechanical behavior of the cornea under uniaxial tensile loading (tensile test) is characterized by higher stresses and strains compared to the inflation test, which simulates physiological conditions with lower stresses but captures the biaxial response of the cornea. Tensile test curves reach stress values up to 7.5 MPa and stretches up to 1.18, indicating a stronger response to uniaxial deformation. In contrast, inflation test curves display much lower stress levels, peaking around 0.05 MPa, with stretches reaching only 1.045. These differences explain why optimized parameters obtained from tensile and inflation tests are not interchangeable. For example, $k_{2}$ values in inflation tests are typically much larger than those in tensile tests.

### Limitations of the Study

This study is subject to some limitations that must be acknowledged. First, the datasets used were limited in number and derived from previously published experimental studies, which may not fully capture the biological variability of corneal tissue. In addition, the study relied on representative smoothed stress–stretch curves from literature instead of raw experimental data, which limits the ability to evaluate the robustness of the optimization process against real experimental noise and variability.

Regarding the modeling assumptions, the inflation test was treated as a spherical shell with uniform thickness, neglecting regional variations in geometry and thickness distribution. While this simplification is common in corneal biomechanics, it inevitably introduces discrepancies with physiological reality.

A particularly relevant limitation concerns the transformation of pressure–apical displacement curves into stress–stretch curves in inflation experiments. This conversion is highly sensitive to the assumed geometric input parameters, such as corneal thickness, apex height, and base radius. Small variations or inaccuracies in these values can significantly alter the computed stress–stretch relationships, thereby affecting the optimized material parameters.

The present analysis also adopts a simplified, general model with fixed geometric inputs rather than subject-specific anatomy; this choice facilitates comparison across datasets but does not capture inter-individual variability in contour, stiffness, and thickness. Finally, the stepwise sequential approach was applied and validated only for the HGO model; although it may extend to models that clearly separate isotropic and anisotropic contributions with few parameters, its applicability to highly coupled multi-parameter laws (e.g., five-parameter Mooney–Rivlin) is limited.

To mitigate these limitations, future work may use raw experimental data rather than smoothed curves; include subject-specific geometry from imaging (apex height, base radius, thickness maps); replace the axisymmetric membrane with a biaxial, non-axisymmetric formulation; add fiber-dispersion and allow non-orthogonal preferred directions; and validate the estimated parameters on independent datasets and in finite-element simulations under physiological intraocular pressure.

## 5. Conclusions

The objectives of this study are to optimize the mechanical parameters of the cornea using both tensile and inflation tests and to address the variability in parameters reported across studies. It is established that different testing methods (tensile and inflation) yield significantly different results in the HGO model parameters $c_{1}$, $k_{1}$, and $k_{2}$, excluding $c_{2}$ to simplify the model. To achieve this, the parameters were optimized across different optimization ranges for three tensile curves with varying degrees of nonlinearity and three inflation curves.

A key factor contributing to the variability across studies includes differences in the specific corneal zones that are tested, and initial geometric measurements like base, height, and radius in the case of inflation tests. Mathematical simplifications in virtual tests also play a role in this variability. However, it is identified and demonstrated as the primary source of variation that multiple sets of parameters can highly correlate with experimental stress–stretch curves while still making physical sense. This presents a challenge in reducing parameter variability.

This study proposes a stepwise approach to mitigate this variability. First, $c_{1}$ should be optimized based on initial deformations from tensile tests, where isotropic stiffness dominates. Once $c_{1}$ is fixed, the anisotropic parameters $k_{1}$ and $k_{2}$, which capture fiber alignment and non-linear stiffening, may be optimized. This method simplifies the optimization process and reduces variability in parameter estimation.

Through a comparative analysis, the study demonstrates that tensile and inflation test data are not interchangeable. Each test yields different parameter values, as the stress–stretch scales differ significantly.

## Figures and Tables

**Table 1:** Estimated HGO material parameters from the tensile tests for three different optimization intervals of the three curves.

*Interval*	*Curve*	$c_{1}$ (MPa)	$c_{2}$ (MPa)	$k_{1}$ (MPa)	$k_{2}$ (−)	$R^{2}$
*Interval 1 (Slightly Restrictive):* *[0, 50], [0, 50], [0, 100], [0, 1000]*	Curve 1 (Highly non-linear)	0.39	0	0.2721	24.656	0.99
	Curve 2 (Moderately non-linear)	1.57	0	0.8743	8.254	0.99
	Curve 3 (Slightly non-linear)	3.74	0	0.6872	9.378	0.97
*Interval 2 (Non-Restrictive):* *[−100, 100], [−100, 100], [0, 100], [0, 1000]*	Curve 1 (Highly non-linear)	12.57	−12.89	0.1911	26.914	0.99
	Curve 2 (Moderately non-linear)	39.89	−40.43	0.3106	10.726	0.99
	Curve 3 (Slightly non-linear)	65.97	−66.05	0.0426	0.750	0.99
*Interval 3 (Highly Restrictive):* *[0.005, 0.05], [−0.05, −0.005], [0, 100], [0, 1000]*	Curve 1 (Highly non-linear)	0.05	−0.005	0.3203	23.504	0.99
	Curve 2 (Moderately non-linear)	0.05	−0.005	1.393	6.318	0.99
	Curve 3 (Slightly non-linear)	0.05	−0.005	2.0899	4.571	0.98

**Table 2:** Estimated HGO material parameters from the inflation tests for three different optimization intervals of the three curves.

*Interval*	*Curve*	$c_{1}$ (MPa)	$c_{2}$ (MPa)	$k_{1}$ (MPa)	$k_{2}$ (−)	$R^{2}$
*Interval 1 (Slightly Restrictive):* *[0, 50], [0, 50], [0, 100], [0, 1000]*	Curve 1	0	0.006	0.0243	223.66	0.99
	Curve 2	0	0	0.0601	229.26	0.99
	Curve 3	0	0	0.0717	434.87	0.99
*Interval 2 (Non-Restrictive):* *[−100, 100], [−100, 100], [0, 100], [0, 1000]*	Curve 1	−0.60	0.6054	0.0054	349.09	0.99
	Curve 2	2.905	−3.792	1.393	28.87	0.99
	Curve 3	1.494	−1.632	0.2917	216.23	0.99
*Interval 3 (Highly Restrictive):* *[0.005, 0.05], [−0.05, −0.005], [0, 100], [0, 1000]*	Curve 1	0.0109	−0.005	0.0251	221.07	0.99
	Curve 2	0.005	−0.05	0.1092	166.47	0.99
	Curve 3	0.005	−0.05	0.1204	333.22	0.99

**Table 3:** Isotropic parameter $c_{1}$ estimated values for the three curves.

*Parameter*	*Curve*	$c_{1}$ (MPa)
*Interval [0, 50]*	Curve 1	0.0198
	Curve 2	0.0361
	Curve 3	0.0624

**Table 4:** Anisotropic parameters $k_{1}$ and $k_{2}$ estimated values, with fixed $c_{1}$ value.

*Interval* (MPa)	*Curve*	$c_{1}$ (MPa)	$k_{1}$ (MPa)	$k_{2}$ (−)	$R^{2}$
*Interval 1 (Slightly Restrictive):* *[0, 50], [0, 50], [0, 100], [0, 1000]*	Curve 1	0.0198	0.0143	273.48	0.99
	Curve 2	0.0361	0.0299	311.86	0.99
	Curve 3	0.0624	0.0226	691.26	0.99

**Table 5:** Corneal parameter data in the scientific literature collected from tensile tests.

*Reference*	$c_{1}$ (MPa)	$c_{2}$ (MPa)	$k_{1}$ (MPa)	$k_{2}$ (−)
*HGO* [11]	0.3312	(−)	60.4949	114.6006
*HGO Pandolfi* [11]	0.3312	(−)	2.9211	20.7395
*HGO* [12]	0.046	(−)	103.5	149.5
[13]	0.0025	(−)	4.115088	5.74966
*M. Nambiar* et al., 2022 [12]	0.021	(−)	87.93	15.2
*Wollensak* et al., 2003 [10]	0.11	−0.01	0.055	14
*Bryant* et al., 1994 [10]	2.11	−2.109	0.2	50
*Hoeltzel* et al., 1992 [10]	15.11	−15.1	0.175	15
*Zeng* et al., 2001 [10]	0.0101	−0.01	0.055	16
*Wollensak* et al., 2003 (MR) [10]	1.11	−1	(−)	(−)
*Hoeltzel* et al., 1992 (MR) [10]	20.01	−20	(−)	(−)

**Table 6:** Corneal parameter data in the scientific literature collected from inflation tests.

Reference	Specimen	$c_{1}$ (MPa)	$c_{2}$ (MPa)	$k_{1}$ (MPa)	$k_{2}$(−)	$c_{1}$ (Mean) (MPa)	$k_{1}$ (New) (MPa)	$k_{2}$ (New)
[15]	1	0.00291	(−)	0.0338	291.28	0.004	0.0339	294.41
	2	0.00357	(−)	0.0219	362.33	0.004	0.0215	368.2
	3	0.04691	(−)	0.0324	1080.51	0.004	0.1054	409.55
	4	0.03744	(−)	0.0217	1280.07	0.004	0.0758	518.76
	5	0.00275	(−)	0.068	435.38	0.004	0.0684	442.72
	6	0.00906	(−)	0.1031	406.51	0.004	0.1049	376.44
	7	0.01605	(−)	0.1602	774.71	0.004	0.1607	664
	8	0.00425	(−)	0.1614	667.85	0.004	0.1609	667.02
	9	0.00328	(−)	0.1482	605.98	0.004	0.1496	607.08
	Mean	0.01402	(−)	0.0834	656.07	0.004	0.0979	483.13
[14]	A	0.05	(−)	25	2490	(−)	(−)	(−)
	B	0.05	(−)	60	2490	(−)	(−)	(−)
	C	0.05	(−)	130.9	2490	(−)	(−)	(−)
[10]	Healthy (Inflation)	0.32	−0.25	0.055	750	(−)	(−)	(−)
	Keratoconus (periphery) (Inflation)	0.32	−0.25	0.005	400	(−)	(−)	(−)
	Keratoconus (center) (Inflation)	0.32	−0.25	0.005	200	(−)	(−)	(−)

## Data Availability

Data will be made available upon request.

## List of Acronyms

HGO	Holzapfel–Gasser–Ogden
a	Base radius of the cornea
$c_{i}(i=1,2)$	Material constant of the Mooney–Rivlin model
h	height of the dome
h_0_	Initial height of the dome
$k_{1}$	Hyperelastic material parameter to consider the influence of collagen fibrils’ stiffness
$k_{2}$	Hyperelastic material parameter to consider the influence of collagen fibrils on large extensions
J	Jacobian of the deformation gradient tensor
b	Left Cauchy–Green deformation tensor
C	Right Cauchy–Green deformation tensor
$e_{R}$	Unidirectional vector in radial direction
$e_{\varphi }$	Unidirectional vector in meridional direction
$e_{\theta }$	Unidirectional vector in circumferential direction
**F**	Deformation gradient tensor
$m_{0}$	Initial preferential orientation of collagen fibril family 1
m	Deformed preferential orientation of collagen fibril family 1
$n_{0}$	Initial preferential orientation of collagen fibril family 2
n	Deformed preferential orientation of collagen fibril family 2
*P*	Pressure
*R*	Radius of curvature
$R^{2}$	Coefficient of determination
*SS_Res_*	Sum of squared errors between the predicted and experimental values
*SS_Tot_*	Sum of square deviations of the experimental values and their average
*t*	Cornea’s thickness
*t*_0_	Initial Cornea’s thickness before pressurization
***x***	Deformed vector
***X***	Reference vector
$\lambda_{i}\;(i=1,\;3)$	Stretch ratio component
$\sigma_{i}\;(i=1,\;3)$	Cauchy stress component
ψ	Strain energy density
$\psi_{deviatoric}$	Deviatoric strain energy density
$\bar{I_{i}}$ (i = 1, 2, 4, and 6)	Invariants of the Right Cauchy Stress Tensor

## Appendix A

To derive the stress–stretch relationship according to [22], the applied pressure is converted into the appropriate units in Pa.

(A1) $$P_{\left(Pa\right)}=P_{\left(mmHg\right)}\times 133.322.$$

The cornea is approximated as a spherical shell, assuming that the applied pressure generates an apical displacement and homogenous surface deformation. The radius of curvature R is calculated as

(A2) $$R=\frac{\left(a^{2}+h^{2}\right)}{2h},$$

(A3) $$h=h_{0}+w,$$

where $h_{0}$ is the initial height of the dome, w is the apical displacement, and a is the base radius of the cornea. This formula assumes the cornea deforms as a spherical cap.

The stretch λ is calculated from the deformed radius of curvature R and the initial radius of curvature before pressurization $R_{0}$.

(A4) $$\lambda =\frac{\left(R\;{sin}^{-1}(a/R)\right)}{\left(R_{0}\;{sin}^{-1}(a/R_{0})\right)}.$$

The stress in the cornea can be approximated using Laplace’s law, which relates the applied pressure $P_{\left(Pa\right)}$, deformed radius R, and the deformed corneal thickness t:

(A5) $$\sigma \;=\;\frac{P_{\left(Pa\right)}R}{2t},$$

(A6) $$t=\frac{t_{0}}{\lambda^{2}},$$

where $t_{0}$ is the initial thickness of the cornea before pressurization.

## References

[1] Burton M.J., Ramke J., Marques A.P., Bourne R.R.A., Congdon N., Jones I., Tong B.A.M.A., Arunga S., Bachani D., Bascaran C. (2021). The Lancet Global Health Commission on Global Eye Health: Vision beyond 2020. Lancet Glob. Health.

[2] Enaholo E.S., Musa M.J., Zeppieri M. (2024). The Spherical Equivalent. StatPearls.

[3] Alkanaan A., Barsotti R., Kirat O., Khan A., Almubrad T., Akhtar S. (2019). Collagen fibrils and proteoglycans of peripheral and central stroma of the keratoconus cornea: Ultrastructure and 3D transmission electron tomography. Sci. Rep..

[4] Liu T., Shen M., Li H., Zhang Y., Mu B., Zhao X., Wang Y. (2020). Changes and quantitative characterization of hyper-viscoelastic biomechanical properties for young corneal stroma after standard corneal cross-linking treatment with different ultraviolet-A energies. Acta Biomater..

[5] Wang J., Liu X.Y., Bao F.J., Lopes B.T., Wang L.Z., Eliasy A., Abass A., Elsheikh A. (2021). Review of ex-vivo characterization of corneal biomechanics. Med. Nov. Technol. Devices.

[6] Santodomingo-Rubido J., Carracedo G., Suzaki A., Villa-Collar C., Vincent S.J., Wolffsohn J.S. (2022). Keratoconus: An updated review. Contact Lens Anterior Eye.

[7] Holzapfel G.A., Gasser T.C., Ogden R.W. (2000). A new constitutive framework for arterial wall mechanics and a comparative study of material models. J. Elast. Phys. Sci. Solids.

[8] Gómez C., Piñero D.P., Peredes M., Alió J.L., Cavas F. (2021). Iterative methods for the biomechanical evaluation of corneal response: A case study in the measurement phase. Appl. Sci..

[9] Pandolfi A. (2020). Cornea modelling. Eye Vis..

[10] Pandolfi A., Manganiello F. (2006). A model for the human cornea: Constitutive formulation and numerical analysis. Biomech. Model. Mechanobiol..

[11] Liu T., Shen M., Huang L., Xiang Y., Li H., Zhang Y., Wang Y. (2020). Characterization of hyperelastic mechanical properties for youth corneal anterior central stroma based on collagen fibril crimping constitutive model. J. Mech. Behav. Biomed. Mater..

[12] Nambiar M.H., Liechti L., Studer H., Roy A.S., Seiler T.G., Büchler P. (2023). Patient-specific finite element analysis of human corneal lenticules: An experimental and numerical study. J. Mech. Behav. Biomed. Mater..

[13] Lanchares E. (2010). Modelado Biomecánico de los Componentes Refractivos del Ojo Humano y Tratamientos Refractivos Asociados. Ph.D. Thesis.

[14] Ariza-Gracia M.Á. (2018). Methods for Characterising Patient-Specific Corneal Biomechanics. Ph.D. Thesis.

[15] Kok S., Botha N., Inglis H.M. (2014). Calibrating corneal material model parameters using only inflation data: An ill-posed problem. Int. J. Numer. Methods Biomed. Eng..

[16] Elsheikh A., Anderson K. (2005). Comparative study of corneal strip extensometry and inflation tests. J. R. Soc. Interface.

[17] Studer H., Riedwyl H., Büchler P. (2011). Importance of multiple loading scenarios for the identification of material coefficients of the human cornea. Comput. Methods Biomech. Biomed. Eng..

[18] Scarcelli G., Besner S., Pineda R., Yun S.H. (2014). Biomechanical characterization of keratoconus corneas ex vivo with Brillouin microscopy. Investig. Ophthalmol. Vis. Sci..

[19] Foong T.Y., Hua Y., Amini R., Sigal I.A. (2023). Who bears the load? IOP-induced collagen fiber recruitment over the corneoscleral shell. Exp. Eye Res..

[20] Hatami-Marbini H., Emu M.E. (2025). Biomechanical properties of porcine cornea; planar biaxial tests versus uniaxial tensile tests. J. Mech. Behav. Biomed. Mater..

[21] Muñoz-Villaescusa C., Núñez-Chongo O.d.l.C., Cárdenas-Díaz T., Batista-Leyva A.J., Cavas-Martínez F. (2021). Experimental Determination of Corneal Elastic Constants and Their Use in Biomechanical Modeling. Appl. Sci..

[22] Roberts C.J., Dupps W.J., Downs J.C. (2018). Biomechanics of the Eye.

